# Epigenetic alterations in autoimmune disease

**DOI:** 10.1186/1479-5876-9-S2-I3

**Published:** 2011-11-23

**Authors:** Esteban Ballestar, Biola M Javierre, Lorenzo de la Rica, David Gómez-Cabrero, Jesper Tegnér, Carmen Gomez-Vaquero, Javier Narvaez, Henar Hernando, Virginia C Rodriguez, Roser Vento, Laura Ciudad, Javier Rodriguez-Ubreva

**Affiliations:** 1 Chromatin and Disease Group, Cancer Epigenetics and Biology Programme (PEBC), Bellvitge Biomedical Research Institute (IDIBELL), L'Hospitalet de Llobregat, Barcelona, Spain; 2 Dept. of Medicine, Karolinska Institutet, Computational Medicine Unit, Centre for Molecular Medicine, and Swedish e-science Research Centre (SeRC), Solna, Stockholm, Sweden; 3 Dept. of Rheumatology, Bellvitge University Hospital, L'Hospitalet de Llobregat, Barcelona, Spain

## Background

In the past years, we have witnessed unprecedented attention for the study of epigenetic alterations in the context of a variety of complex disorders, including autoimmune diseases [1]. This is in part due to the observation that genetics is insufficient to entirely explain the predisposition to their pathogenesis. The environmental influence is well illustrated by the existence of partial concordance for susceptibility to disease in monozygotic twins. In connection with this epigenetic mechanisms regulate gene expression and are sensitive to external stimuli, bridging the gap between environmental and genetic factors. There is now considerable evidence of the existence of epigenetic alterations, particularly DNA methylation alterations, in diseases like systemic lupus erythematosus or rheumatoid arthritis [1]. Most of the studies were initially performed by using candidate-gene approaches, although the increasing availability of high-throughput methods is providing better methods for the screening of epigenetic alterations in these diseases [2].

## Materials and methods

We have used different methylation bead arrays to profile the DNA methylation status of CpG sites in both samples obtained from patients from different autoimmune diseases and normal individuals as well as different experimental models that allow to explore conditions where the immune tolerance is lost. Following bioinformatics analysis, we individually validate relevant genes by bisulfite pyrosequencing and check their expression levels by quantitative RT-PCR. The potential role of different chromatin factors in these changes is then explored by using experimental models where these factors can be overexpressed or knocked-down. Also, their direct association at specific genomic sites can be investigated by using chromatin immunoprecipitation assays.

## Results

High-throughput and candidate sequence analysis of DNA methylation in samples from different autoimmune diseases show the existence of alterations associated with these diseases. We have also identified DNA methylation changes in association with several factors associated with autoimmune disease like infection with Epstein Barr-virus of B cells. Most of the changes observed occur in the direction towards hypomethylation associated with gene upregulation.

## Conclusions

Our findings are providing potentially relevant DNA methylation markers for the clinical managements of different autoimmune diseases and also support the notion that epigenetic alterations may be critical in the pathogenesis of autoimmune disease.
